# Protocol for the detection of RHIM-protein oligomeric complex formation using DSP-mediated crosslinking in HEK293T cultured cells

**DOI:** 10.1016/j.xpro.2025.104190

**Published:** 2025-11-06

**Authors:** Sanchita Mishra, Ayushi Amin Dey, Sannula Kesavardhana

**Affiliations:** 1 Department of Biochemistry, Division of Biological Sciences, Indian Institute of Science, Bengaluru, Karnataka 560012, India

**Keywords:** Immunology, Microbiology, Molecular Biology, Protein Biochemistry

## Abstract

Proteins harboring the RIP homotypic interaction motifs (RHIMs) can assemble into higher-order structures through RHIM-RHIM interactions. Here, we present a protocol for detecting RHIM protein oligomerization, which is central to cell death and inflammatory signaling. We describe the steps for expressing RHIM proteins in HEK293T cells, crosslinking their oligomers, and detecting them by immunoblotting analysis. This protocol can facilitate the study of RHIM-based oligomerization and its role in modulating cell death and inflammation.

For complete details on the use and execution of this protocol, please refer to Mishra et al.^1^

## Highlights

• Steps for RHIM protein expression in HEK293T and detection of RHIM oligomers
• Guidance for DSP-mediated crosslinking of interacting RHIM proteins
• Instructions to stabilize RHIM-protein interactions and their oligomeric complexes

## Abstract (editor highlights)

Publisher’s note: Undertaking any experimental protocol requires adherence to local institutional guidelines for laboratory safety and ethics.

## Graphical abstract

**Figure:**
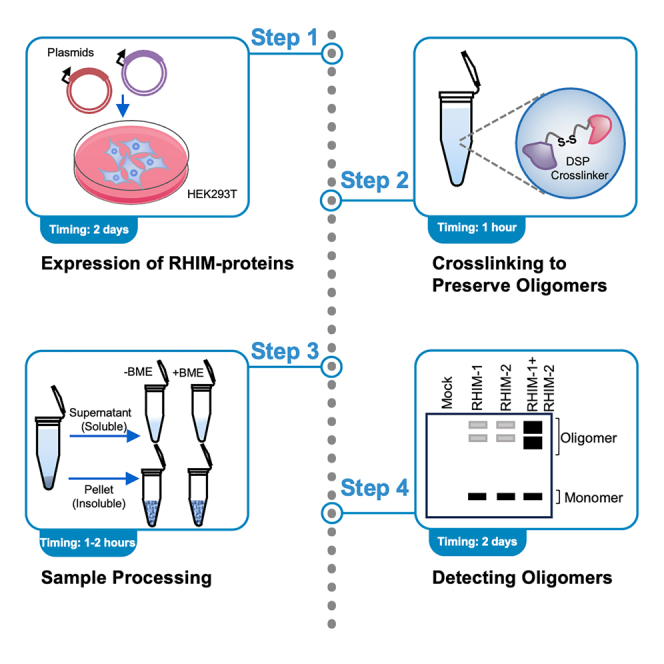

## Before you begin

**Figure 1:**
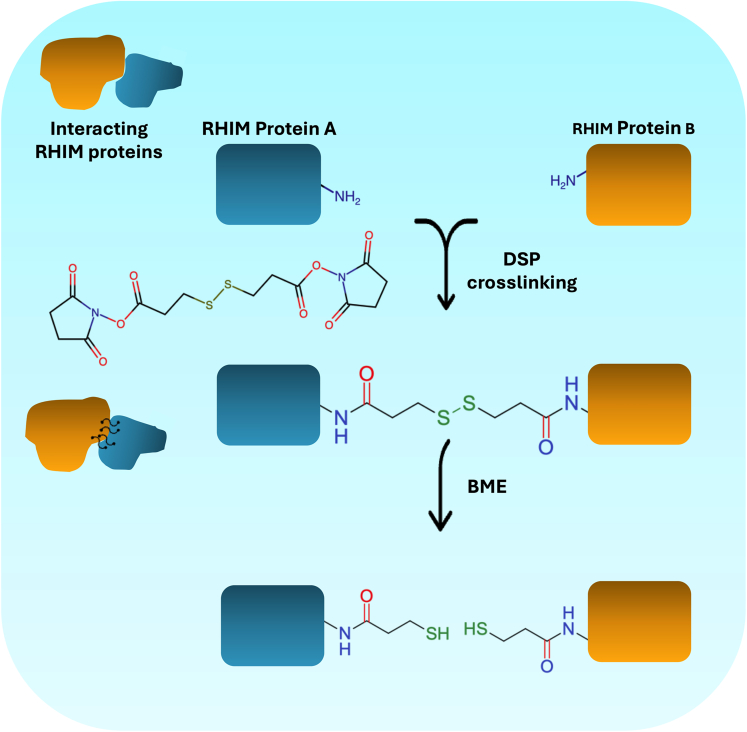
Mechanism of action for DSP-mediated crosslinking of interacting RHIM proteins DSP (Dithiobis (succinimidyl propionate)) reacts with the proximal amine groups of the RHIM proteins to crosslink them. The disulfide in the spacer region of DSP enables the crosslinked proteins to be decoupled using a reducing agent like BME (β-mercaptoethanol). The chemical backbones were drawn using ChemAxon’s Marvin JS structure drawing tool.

Regulated inflammatory cell death activation in response to pathogenic infections or endogenous triggers is an innate host defense mechanism.^2^^,^^3^^,^^4^ Receptor-interacting serine/threonine protein (RIP) kinase 1 (RIPK1), RIPK3, Toll/IL-1 receptor domain-containing adapter protein-inducing IFN-β (TRIF), and Z-nucleic acid binding protein 1 (ZBP1) are cell death signaling proteins that harbor conserved protein motifs called RIP Homotypic interaction motifs (RHIM).^5^^,^^6^ RHIMs mediate homotypic protein-protein interactions and oligomerization of the cell death proteins. The core tetrad residues (I/V-Q-I/V/L/C-G) in RHIMs are critical for stacking β-sheet structures to form higher-order amyloid-like signaling complexes, essential for cell death and inflammatory signaling.^6^^,^^7^^,^^8^ Several viruses encode viral RHIM proteins that mimic host RHIMs and modulate host cell death.^9^^,^^10^^,^^11^ Here, we describe a protocol for detecting RHIM-mediated complex formation of host/viral RHIM proteins. The protocol involves DSP-mediated crosslinking to preserve the oligomeric complexes. DSP (dithiobis(succinimidylpropionate)) is a membrane-permeable crosslinking reagent that reacts with the primary amines of interacting proteins to crosslink them. The crosslinked complexes can be decoupled with reducing agents due to an intrinsic disulfide bond in the spacer arm of DSP (Figure 1). This protocol will be useful for the quick and easy detection of RHIM-mediated complex formation. It will further provide a better understanding of RHIM-RHIM interaction-mediated host cell death signaling and the crosstalk between host and viral RHIM proteins. Using this protocol, we recently demonstrated that the viral RHIM (CoV-RHIM-1) in SARS-CoV-2 Nsp13 promotes higher order complex formation of host RHIM proteins - ZBP1 and RIPK3, when co-expressed in HEK-293T cells, to promote cell death.^1^

### Innovation

RHIM containing proteins are known to aggregate into amyloid structures and often rapidly precipitate. This property makes the study of RHIM-driven complexes of full length RHIM proteins quite challenging. The visualization of RHIM-based amyloid assemblies largely relies on the purification of specific protein fragments containing the RHIM domain and further in vitro assays. While these assays have been quite useful in understanding the dynamics of RHIM protein assembly, they do not represent the true cellular environment and additional mechanisms other than RHIM-interaction are not taken into consideration. Our simple cell-based assay is a critical tool to examine cellular RHIM-protein oligomerization in a biologically relevant system while preserving the effect of other cellular processes and factors influencing this assembly. DSP mediated cross-link can stabilize the transient or weak interactions between the RHIM proteins, which might dissociate during cell lysis. We have employed this biochemical assay to demonstrate for the first time, the oligomerization of host RHIM - ZBP1 in the presence of a novel RNA viral RHIM – Nsp13. This method circumvents many limitations of existing methods and is a critical tool for performing functional studies on RHIM protein oligomerization and its effect on cell fate decisions under various stress conditions.

## Key resources table

**Table:** 

REAGENT or RESOURCE	SOURCE	IDENTIFIER
**Antibodies**		

Mouse monoclonal Anti-ZBP1 (Zippy-1) (dilution used: 1:1,000)	AdipoGen Life Sciences	AG-20B-0010-C100
Mouse monoclonal Anti-Strep-Tag (dilution used: 1:1,000)	QIAGEN	34850; RRID:AB_2810987
Mouse monoclonal anti-HA tag (dilution used: 1:1,000)	Invitrogen	26183; RRID:AB_2533049
Peroxidase AffiniPure rabbit anti-mouse IgG (dilution used: 1:5,000)	Jackson ImmunoResearch	315-035-047; RRID:AB_2340068

**Chemicals, peptides, and recombinant proteins**		

DMEM, high glucose, pyruvate	Thermo Fisher Scientific	11995081
Antibiotic-antimycotic	Thermo Fisher Scientific	15240062
Fetal bovine serum (FBS)	Thermo Fisher Scientific	10270106
Phosphate-buffered saline (PBS)	Sigma-Aldrich	D5652
Trypsin (2.5%), no phenol red	Thermo Fisher Scientific	15090046
Non-essential amino acids	Thermo Fisher Scientific	11140-050
Xfect transfection reagent	Takara Bio	631318
Opti-MEM (reduced serum media)	Thermo Fisher Scientific	31985070
Pierce Premium Grade DSP	Thermo Fisher Scientific	PG82081
Immobilon Forte western HRP substrate	Millipore	WBLUF0500
PVDF blotting membrane	Cytiva	10600023
HEPES (1 M)	Thermo Fisher Scientific	15630080
NP-40 alternative	Millipore	492016
Tris base	Qualigens	Q15965
Glycine	Qualigens	Q24755
Sodium chloride	Qualigens	Q27605
Sodium dodecyl sulfate	Sigma-Aldrich	L3771
Acrylamide	Sigma-Aldrich	A8887
Bis-acrylamide (N,N′-methylenebisacrylamide)	Sigma-Aldrich	M7279
Ammonium persulfate (APS)	Sigma-Aldrich	A3678
N,N,N′,N′-tetramethylethylenediamine (TEMED)	Sigma-Aldrich	T7024
Glycerol	Sigma-Aldrich	G5516
Bromophenol blue dye	Sigma-Aldrich	B0126
Phosphatase inhibitor (PhosSTOP)	Sigma-Aldrich	PHOSSRO
cOmplete, Mini, EDTA-free protease inhibitor cocktail	Sigma-Aldrich	11836170001
Methanol	Qualigens	Q3445C
Isopropanol	Qualigens	Q26895
Tween 20	Sigma-Aldrich	P1379
Skimmed milk powder	HiMedia	GRM1254
β-mercaptoethanol	Sigma-Aldrich	M3148

**Experimental models: Cell lines**		

HEK293T (human embryonic kidney)	National Centre for Cell Science (NCCS) cell repository	N/A

**Recombinant DNA**		

pLVX-EF1alpha-SARS-CoV-2-Nsp13-2xStrep-IRES-Puro	Nevan Krogan Lab https://doi.org/10.1038/s41586-020-2286-9	N/A
pLVX-EF1alpha-SARS-CoV-2-Nsp13-Tet-Mut-2xStrep-IRES-Puro	This Paper	N/A
pcDNA3-HA-RIPK3	Seo et al., https://doi.org/10.1038/ncb3314	78804; Addgene plasmid
pCMV-Human-ZBP1 cDNA	Sino Biological	HG19385-UT

**Software and algorithms**		

ImageJ 1.54p	NIH	https://imagej.nih.gov/ij/

**Other**		

ImageQuant LAS500 or ImageQuant 800	Cytiva, Amersham	

## Materials and equipment

**Table:** NP-40 HEPES crosslinking buffer

Reagent	Final concentration	Amount
HEPES Buffer	50 mM	2.5 mL
NaCl solution(5 M)	150 mM	1.5 mL
NP-40	1%	500 μL
Protease inhibitor	N/A	1 tablet
Phosphatase inhibitor	N/A	2 tablets
Distilled Water	N/A	Up to 50 mL
**Total**	**N/A**	**50 mL**

Make fresh before use. Store HEPES buffer, NaCl stock solution, protease and phosphatase inhibitors at 4°C. Store NP-40 at 25°C.

***Note:*** Crush the protease and phosphatase inhibitor tablets properly to dissolve in the buffer.

**CRITICAL:** Add DSP stock solution (100 mM) to the NP-40 HEPES crosslinking buffer at a working concentration of 2 mM just before adding lysis buffer to the cells. Pipette NP-40 slowly and carefully as it is viscous.

**Table:** Stop solution (1 M Tris-Cl, pH 8)

Reagent	Final concentration	Amount
Tris base	1 M	242.28 mg
Distilled Water	N/A	Up to 2 mL
**Total**	**N/A**	**2 mL**

Store at 4°C for 3-4 weeks.

***Note:*** Adjust the pH of the solution to 8 using concentrated HCl (32.36 M).

**Table:** 4X Reducing sample buffer

Reagent	Final concentration	Amount
1 M Tris-Cl, pH 6.8	200 mM	6 mL
SDS	277.41 mM	2.4 g
Bromophenol Blue	0.01%	3 mg
Glycerol	40%	12 mL
β-Mercaptoethanol	2.8%	0.84 mL
Distilled Water	N/A	Up to 30 mL
**Total**	**N/A**	**30 mL**

Store at 20°C–25°C indefinitely.

**Table:** 4X non-reducing sample buffer

Reagent	Final concentration	Amount
1 M Tris-Cl, pH 6.8	200 mM	6 mL
SDS	277.41 mM	2.4 g
Bromophenol Blue	0.01%	3 mg
Glycerol	40%	12 mL
Distilled Water	N/A	Up to 30 mL
**Total**	**N/A**	**30 mL**

Store at 20°C–25°C indefinitely.

***Note:*** Always pipette glycerol slowly and carefully, as it is viscous. Remove the tip from the liquid only when additional liquid stops entering the tip. Also, release the pipette slowly while dispensing glycerol, taking care that most of the liquid leaves the tip. Then pipette up and down multiple times, ensuring that you wash out any residual glycerol in the tip.

**Table:** 29% Acrylamide-1% bis-acrylamide solution

Reagent	Final concentration	Amount
Acrylamide	29%	290 g
bis-acrylamide	1%	10 g
Distilled Water	N/A	Up to 1 L
**Total**	**N/A**	**1 L**

Mix the components well by swirling. Protect the solution from light and store the solution at 4°C for up to 6 months.

**Table:** 8% SDS resolving gel

Reagent	Final concentration	Amount
29% Acrylamide-1% bis-acrylamide	8%	1.3 mL
1.5 M Tris-Cl, pH 8.8	390 mM	1.3 mL
10% SDS	0.1%	50 μL
10% APS	0.1%	50 μL
TEMED	0.06%	3 μL
Distilled Water	N/A	Up to 5 mL
**Total**	**N/A**	**5 mL**

Make fresh just before use. Store distilled water, Tris-Cl stock solution, TEMED and 10% SDS solution at 20°C–25°C. Store all other stock solutions at 4°C.

***Note:*** This volume is for one gel. Scale up proportionally. (The dimensions of one gel are 8.6 X 6.7 X 0.1; Width X Length X Thickness in cm).

**Table:** SDS stacking gel solution

Reagent	Final concentration	Amount
29% Acrylamide-1% bis-acrylamide	16.5%	330 μL
1 M Tris-Cl, pH 6.8	125 mM	250 μL
10% SDS	0.1%	20 μL
10% APS	0.1%	20 μL
TEMED	0.1%	2 μL
Distilled Water	N/A	Up to 2 mL
**Total**	**N/A**	**2 mL**

Make fresh just before use. Store distilled water, Tris-Cl stock solution, TEMED and 10% SDS solution at 20°C–25°C. Store all other stock solutions at 4°C.

**Table:** 10X SDS running buffer

Reagent	Final concentration	Amount
Tris base	24.76 mM	30.3 g
Glycine	1.92 M	144 g
SDS	3.47 mM	10 g
Distilled Water	N/A	Up to 1 L
**Total**	**N/A**	**1 L**

Store at 20°C–25°C indefinitely.

**Table:** 10X Transfer buffer

Reagent	Final concentration	Amount
Tris base	62.32 mM	75.5 g
Glycine	4.77 M	360 g
Distilled Water	N/A	Up to 1 L
**Total**	**N/A**	**1 L**

Store at 20°C–25°C indefinitely.

**Table:** 1X Transfer buffer

Reagent	Final concentration	Amount
10X Transfer buffer	1X buffer	50 mL
Methanol	20%	100 mL
Distilled Water	N/A	Up to 500 mL
**Total**	**N/A**	**500 mL**

Make fresh just before use.

**Table:** 10X Wash buffer

Reagent	Final concentration	Amount
Tris base	20 mM	24.228 g
NaCl	1.48 M	86.66 g
Distilled Water	N/A	Up to 1 L
**Total**	**N/A**	**1 L**

Store at 20°C–25°C indefinitely.

***Note:*** Adjust the pH of the solution to 7.4 using concentrated HCl (32.36 M).

**Table:** 1X Wash buffer

Reagent	Final concentration	Amount
10X Wash buffer	1X	100 mL
Tween-20	0.05%	500 μL
Distilled Water	N/A	Up to 1 L
**Total**	**N/A**	**1 L**

Make fresh just before use.

**Table:** Blocking buffer

Reagent	Final concentration	Amount
Skimmed milk powder	5%	2.5 g
1X Wash buffer	N/A	50 mL
**Total**	**N/A**	**50 mL**

Make fresh just before use by swirling and dissolving the powder in 1X Wash buffer.

**Table:** DSP stock solution

Reagent	Final concentration	Amount
DSP	100 mM	1 g
DMSO	N/A	24.72 mL
**Total**	**N/A**	**24.72 mL**

Aliquot the stock solution into multiple vials and store them at −80°C.

## Step-by-step method details

### Expressing RHIM proteins in HEK293T cells

**Timing: 2 days**

HEK293T cells do not express endogenous ZBP1 and RIPK3. In this step, we transfect HEK293T cells with plasmids for expressing host RHIM proteins (ZBP1 and RIPK3) and viral RHIM protein (SARS-CoV-2 Nsp13).

1. Seed HEK293T cells in 35 mm or 100 mm dishes.
  ***Note:*** Check the confluency and morphology of HEK293T cells daily to ensure they are healthy before seeding. Use cells in the early passages for the experiment. Troubleshooting 1.
  a. Remove the culture medium from the T75 flask containing confluent HEK293T cells.
  b. Rinse the cells gently with 5 mL PBS and discard.
  c. Add 2 mL of 0.25% trypsin.
  d. Swirl the flask gently to evenly distribute the trypsin.
  e. Incubate the flask at 37°C and 5% CO_2_ for 30–60 seconds.
  f. Once the cells start detaching, add 5 mL of complete DMEM to the trypsin-treated cells.
  g. Harvest the cells in a 15 mL centrifuge tube.
    ***Note:*** Volumes of PBS, trypsin and complete DMEM mentioned are for cells grown in a T-75 flask.
  h. Spin the cell suspension at 45 *x g* (500–600 rpm) for 3 minutes.
  i. Discard the supernatant.
  j. Resuspend the cell pellet using 1-2 mL of complete DMEM media.
  k. Count the cells using an automated cell counter or hemocytometer.
  l. Seed 0.23 x 10^6^ cells per 35 mm dish or 2 x 10^6^ cells per 100 mm dish in 2 mL or 7 mL complete DMEM respectively.
    ***Note:*** We seeded and transfected the cells in 35 mm dishes for this experiment ensuring the use of smaller quantities of all subsequent components. As we could recapitulate the results with both 35 mm and 100 mm dishes, we suggest using 35 mm dishes because it is economical and easy to handle.
  m. Swirl the dish slowly and uniformly to ensure even distribution of cells in the dishes.
  n. Incubate cells for 20–24 hours at 37°C and 5% CO_2_.
    ***Note:*** Scale the cell numbers up or down, based on seeding conditions.
2. After 20-24 hours of incubation, transfect the HEK293T cells at 60%–70% confluency, with plasmid constructs expressing the RHIM-proteins (Viral RHIM protein: SARS-CoV-2 Nsp13, Host RHIM proteins: ZBP1, RIPK3). Troubleshooting 2.
  a. Prepare the transfection mix. Troubleshooting 3, Troubleshooting 4.
    i. Thaw the transfection reagent (Xfect polymer) before use.
    ii. Vortex the transfection reagent thoroughly.
    iii. For a 35 mm dish, add 100 μL of Xfect Reaction Buffer to a 1.5 mL microcentrifuge tube. For a 100 mm dish, add 250 μL of Xfect Reaction Buffer to a 1.5 mL microcentrifuge tube.
    iv. For a 35 mm dish, add 1.5 μg of each plasmid in the buffer. For a 100 mm dish, add 3 μg of each plasmid in the buffer.
      ***Note:*** We have used plasmids for transfection in 35 mm dishes in the following amounts.
      Single plasmid (Nsp13/ZBP1/RIPK3) – 1.5 μg.
      Two plasmids (ZBP1+RIPK3/Nsp13+RIPK3/Nsp13+ZBP1) – 1.5 μg + 1.5 μg (3 μg).
    v. Add Xfect polymer (0.3 μL for each μg of plasmid) to the plasmid mix.
    vi. Vortex at high speed to mix them well.
    vii. Incubate the mix at 20°C–25°C for 10 – 15 minutes.
      **CRITICAL:** The incubation of the plasmid and buffer mix at 20°C–25°C for 10–15 min is crucial for the formation of Xfect-DNA nanoparticles complex which allow efficient transfection.
  b. Remove the culture medium from the dishes.
  c. Add 900 μL of opti-MEM to 35 mm dishes or 5 mL of opti-MEM to 100 mm dishes.
  d. After 10–15 minutes of incubation, add the transfection mix dropwise to the cells.
  e. Incubate transfected cells at 37°C and 5% CO_2._
  f. Replace opti-MEM with complete DMEM 6-7 hours after transfection.
  g. Incubate dishes at 37°C and 5% CO_2_ until they are ready for lysis.
    ***Note:*** Xfect polymer is stored at −20°C.
    ***Note:*** Xfect polymer is cytotoxic. Stick to the recommended working concentrations. Refer to the manufacturer’s protocol for details.
    ***Note:*** Include a negative control of untransfected cells which is also subjected to media change (not expressing the protein of interest).

### Cell lysis and DSP-mediated crosslinking

**Timing: 1 h**

In this step, we lyse the cells and perform DSP mediated crosslinking to stabilize the protein-protein interactions driving RHIM protein (ZBP1, RIPK3 or Nsp13) oligomerization.

3. Once the transfected cells start undergoing cell death (roughly 28–30 hours post-transfection), perform cell lysis and DSP-mediated crosslinking. Troubleshooting 3.
  ***Note:*** The cells undergoing cell-death will appear to be floating and morphologically round.
  a. Make the crosslinking NP40 lysis buffer by adding 100 mM DSP such that the final concentration of DSP is 2 mM. Troubleshooting 5.
  b. Remove the culture medium.
  c. Rinse cells with PBS.
  d. Add 237.5 μL of ice-cold crosslinking NP40 buffer to 35 mm dishes.
    Add 1 mL of ice-cold crosslinking NP40 buffer to 100 mm dishes.
  e. Let it stand for around 1 minute.
  f. Collect all the cells in the dish using a cell scraper.
  g. Transfer the lysates into a 1.5 mL microcentrifuge tubes.
  h. Incubate the samples on ice for 45 minutes. Invert mix the samples in intervals of 10 minutes during incubation.
4. Stop DSP-crosslinking process.
  a. Add the STOP solution (1 M Tris-HCl, pH 8) to a final concentration of 50 mM into each sample in the MCTs. We add 12.5 μL of the STOP solution to make the total volume up to 250 μL.
  b. Incubate the samples at 20°C–25°C for 5–7 minutes. Invert mix the samples intermittently.

### Sample preparation

**Timing: 1–2 h**

RHIM proteins are known to assemble into amyloid like aggregates and are often seen to precipitate out of the solution. In this step, we separate the crosslinked lysates into soluble (supernatant) and insoluble (pellet) fractions to understand the nature of oligomers formed by ZBP1, RIPK3 and Nsp13.

5. After the DSP-crosslinking reaction has been stopped, centrifuge the whole cell lysates at 6000 *x g* for 15–20 minutes at 4°C to separate the insoluble fraction (pellet) from the soluble fraction (supernatant).
6. After centrifugation, collect the soluble fraction into a fresh microcentrifuge tube.
7. Prepare the soluble lysate for SDS PAGE –
  a. Divide the supernatant into two equal parts, each part consisting of 125 μL.
  b. Mix one part of lysate with 4X sample buffer with BME (β-mercaptoethanol).
  c. Mix the other part of the lysate with 4X sample buffer without BME. Troubleshooting 5.
8. Prepare the insoluble lysate for SDS PAGE –
  a. Dissolve the pellet with 250 μL NP-40-HEPES lysis buffer (without DSP).
  b. Divide the lysate into two equal parts, each part consisting of 125 μL.
  c. Mix one part of lysate with 4X sample buffer with BME.
  d. Mix the other part with 4X sample buffer without BME. Troubleshooting 5.
9. Boil the samples at 95°C for 5–10 minutes.

### Detecting RHIM-protein oligomers by western blotting

**Timing: 2 days**

In this step, we will resolve the RHIM protein oligomers by SDS-PAGE and analyze the oligomers of different proteins (ZBP1, RIPK3 and Nsp13) using Western blotting.

10. Perform SDS gel electrophoresis.
  a. Prepare 8% SDS PAGE gels with 15 wells.
  b. Assemble the electrophoresis apparatus with the SDS-PAGE gels.
    ***Note:*** We use Mini-PROTEAN Tetra Cell (Bio-Rad) gel electrophoresis system according to the manufacturer’s protocol.
    Electrophoresis apparatus design may differ for other systems.
  c. Fill the apparatus with 1X SDS running buffer.
  d. Remove the combs gently.
  e. Load 18 μL of each sample into the respective wells. Load protein marker.
  f. Run the gels at 80 V until the samples cross the stacking gel and enter the resolving gel.
  g. Increase the voltage to 120 V and run the gels until the 50 kDa marker reaches the bottom of the gel. Troubleshooting 5.
    ***Note:*** If you measure protein concentrations, you can load 15-25 μg of protein into each well.
    ***Note:*** We load the same samples in 3 gels and probe them for Nsp13 (Strep-Tag), ZBP1 and RIPK3 (HA-Tag).
11. Perform semi-dry transfer. Troubleshooting 4.
  a. While the gel is running, activate PVDF membrane in methanol and equilibrate in 1X transfer buffer.
  b. Soak the blot papers in 1X transfer buffer.
  c. Extract the gel from the glass plates after the run is complete and equilibrate in 1X transfer buffer.
  d. Assemble the transfer apparatus.
    ***Note:*** We use Trans-Blot SD Semi-Dry Electrophoretic Transfer Cell (Bio-Rad) according to the manufacturer’s protocol.
  e. Transfer proteins at 22 V for 40 mins.
12. Incubate the PVDF membrane in blocking buffer for 1 hour with gentle rocking.
13. Incubate the membranes with the respective primary antibodies diluted in blocking buffer (1:1000) – anti-Strep-Tag, anti-HA-Tag and anti-ZBP1, at 4°C for 10–14 hours with gentle rocking. Troubleshooting 4.
14. After incubation, wash the membranes with wash buffer for 10 minutes. Repeat this step thrice.
15. Incubate the membrane with HRP-conjugated secondary antibody diluted in blocking buffer (1:5000) at 20°C–25°C for 45–60 min with gentle rocking. Troubleshooting 4, Troubleshooting 6.
16. Wash the membranes with wash buffer for 10 minutes. Repeat this step thrice. Troubleshooting 6.
17. Incubate the membranes with substrate and capture chemiluminescence signals. Troubleshooting 6.

## Expected outcomes

**Figure 2:**
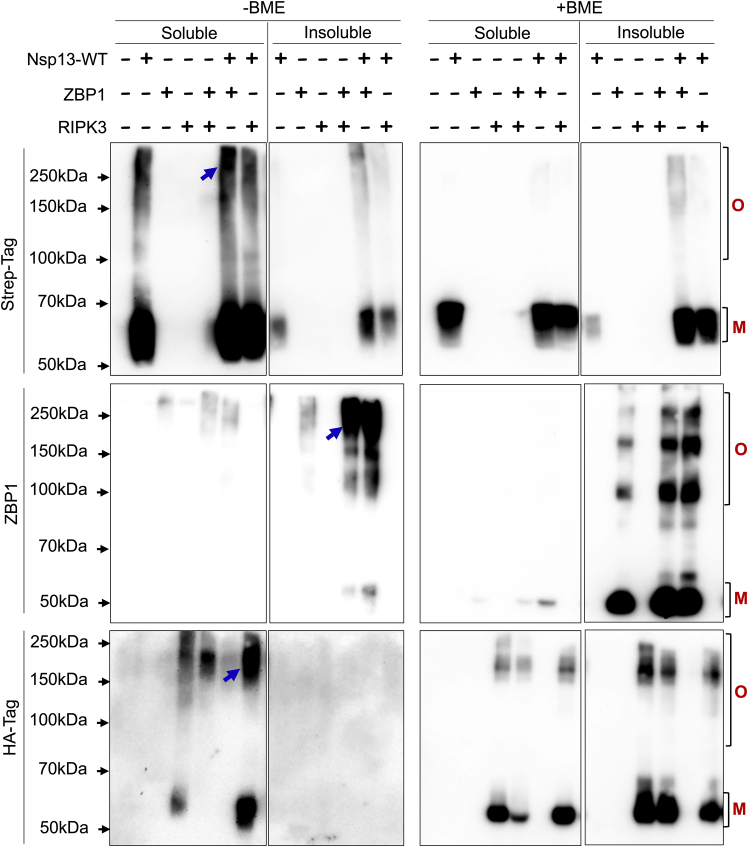
RHIM-RHIM interactions facilitate the assembly of higher-order complexes, preserved by DSP-mediated crosslinking Immunoblot analysis of DSP crosslinked lysates of HEK-293T cells expressing Nsp13-WT, RIPK3, and ZBP1 individually or co-expressing Nsp13-WT+ZBP1, Nsp13-WT+RIPK3, or ZBP1+RIPK3, in non-reduced (without BME) and reduced (with BME) conditions. O- oligomer complexes; M-monomer. The blue arrows indicate oligomer complexes.

RHIM-dependent interactions drive the assembly of large signaling complexes like necrosome and ripoptosome for the activation of necroptosis and apoptosis.^5^^,^^6^^,^^12^^,^^13^ During viral infections, ZBP1 and RIPK3 associate via their RHIMs to form complexes that facilitate virus-induced cell death signaling.^14^^,^^15^^,^^16^ In our protocol, upon crosslinking the cell lysates using DSP, we detect oligomeric complexes of the RHIM proteins (Figure 2). As DSP has a disulfide bond in the spacer region, the crosslinked oligomeric complexes can be dissolved upon treatment with a reducing agent like β-mercaptoethanol (BME). The RHIM-proteins have earlier been reported to self-assemble into homo-oligomers. The expression of ZBP1 and RIPK3 alone in HEK293T cells leads to the formation of low levels of higher-order oligomers of ZBP1 and RIPK3, respectively. As expected, when ZBP1 and RIPK3 are co-expressed, we detect enhanced oligomerization of ZBP1 in the NP40-insoluble fraction. We also observe NP40-soluble and insoluble RIPK3 oligomers. The presence of both ZBP1 and RIPK3 oligomers in the co-expressed samples indicates the presence of hetero-oligomeric assemblies. While these oligomers are largely dissolved into monomers upon treatment with BME, a fraction of the complexes are still retained, suggesting the formation of large insoluble amyloid-like complexes.

**Figure 3:**
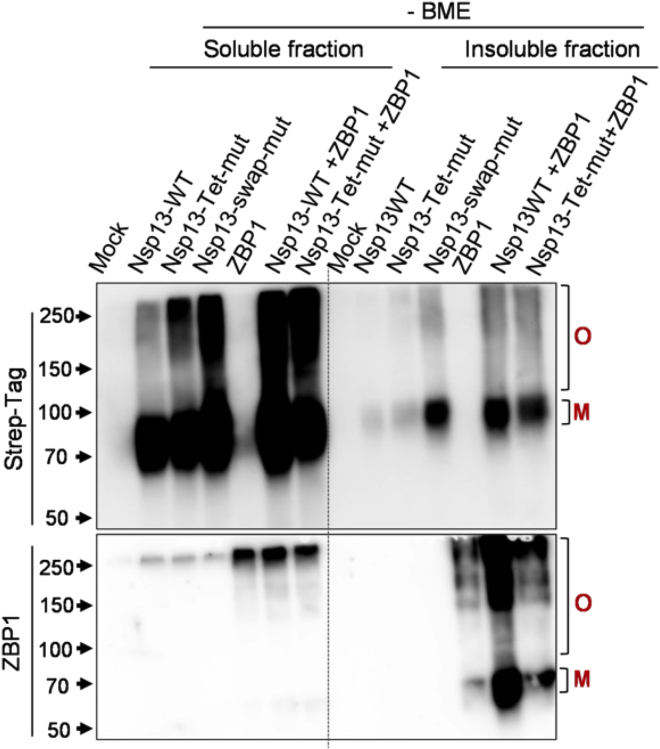
SARS-CoV-2 Nsp13 promotes RHIM-dependent assembly of ZBP1 Immunoblot analysis of crosslinked lysates of HEK-293T cells (soluble and insoluble fractions) expressing SARS-CoV-2 Nsp13-WT, Nsp13-Tet-mut, ZBP1 or co-expressing Nsp13-WT + ZBP1 and Nsp13-Tet-mut + ZBP1. O- oligomer complexes; M-monomer. Figure 3 reprinted and adapted with permission from Mishra et al, 2024.^1^

Several viral proteins have been reported to mimic host RHIMs and promote RHIM-mediated assembly of amyloid complexes by ZBP1, RIPK3, and RIPK1.^6^^,^^17^ The M45 protein from murine cytomegalovirus (MCMV) employs its viral RHIM to form homo-oligomers or heteromeric complexes with RIPK3, RIPK1 and ZBP1. We identified a viral RHIM in SARS-CoV-2 Nsp13 (CoV-RHIM-1) and demonstrated its RHIM-dependent association with ZBP1, leading to cell death in human cells.^1^ This crosslinking protocol is also a tool to monitor the viral modulation of host RHIM complex assembly. We observe that Nsp13 expression alone shows the predominantly monomeric nature of the protein, and we detect some NP-40 soluble oligomers (Figure 2). We have previously demonstrated the RHIM-dependent interaction between Nsp13 and ZBP1. The co-expression of Nsp13 with ZBP1 enhances Nsp13 oligomeric complex formation in the soluble fraction and low levels of oligomers in the insoluble fraction. In accordance with the interaction of Nsp13 with ZBP1, co-expression of ZBP1 with Nsp13-WT significantly enhances the levels of NP40 insoluble ZBP1 oligomers. Similarly, we detect higher-order complexes of RIPK3 upon the co-expression of RIPK3 with Nsp13 (Figure 2). The core tetrad residues of the RHIMs are critical for their interaction and oligomerization. Mutation of the tetrad to AAAA is known to abolish the assembly of RHIM proteins into complexes. In our assay, the magnitude of ZBP1 complexes is reduced when co-expressed with Nsp13-Tet-Mut (mutated CoV-RHIM-1: VQIG → AAAA), suggesting that the assembly of oligomers is RHIM-dependent (Figure 3). These results confirm that our protocol is working for the visualization of RHIM protein oligomers.

## Limitations

This assay biochemically captures higher-order complexes of RHIM-proteins, indicating the cell death activation mechanisms. A potential limitation with this assay is that it does not elucidate the details of homotypic or heterotypic interactions of RHIM proteins. If the readers are interested in the details of the nature of interactions, we suggest performing some additional assays to complement our assay and address the limitation. Immunoprecipitation of the crosslinked complexes using antibodies against the respective RHIM proteins (ZBP1, RIPK3 or Nsp13), followed by mass spectrometric analysis of the samples will reveal the enrichment of either a single RHIM protein (homotypic RHIM interaction) or one RHIM protein with the other (heterotypic RHIM interaction) in the complex. Using crosslinking mass spectrometric analysis, the linkage sites can also be delineated. Our assay cannot exclude the possibility of having non-RHIM proteins in the RHIM-RHIM complexes. The presence of non-RHIM interacting partners in the complexes can also be confirmed and identified by performing Co-IP followed by mass spectrometry.

Alternatively, the readers can purify specific RHIM containing regions of the respective proteins (as reported in multiple studies) to study whether the oligomers contain homomeric or heteromeric assemblies. RHIM proteins are known to self-assemble. Protein samples of individual RHIM-containing constructs (ZBP1, RIPK3 or Nsp13) or mixtures of these could be incubated, crosslinked (optional) and samples can be prepared in reducing or non-reducing conditions. We recommend analyzing the samples by SDS-PAGE and Western blotting. The presence and extent of oligomerization in the lanes loaded with samples containing individual or mixture of proteins will reveal how homotypic or heterotypic interactions affect higher order assembly of these proteins. We suggest the incorporation of controls like a RHIM-dead mutant to confirm that the interactions are RHIM-dependent.

## Troubleshooting

### Problem 1

Poor cell health before seeding for experiment. Protocol major step 1.

### Potential solution

• Follow a good cell maintenance protocol for optimal cell health. Do not let cells become overconfluent before passaging the cells. Do not subculture cells when cell density is too low.
• Do not leave the cells in trypsin for longer than 30–60 seconds.
• Do not agitate the cells during collection. Collect gently using a serological pipette.
• Be gentle while resuspending the cell pellet to avoid cell stress.
• Change media whenever required to maintain optimal pH and nutritional requirements.

### Problem 2

Cell morphology is unhealthy 24 hours post-seeding. Protocol major step 1.

### Potential solution

• Cells are being cultured for too many passages. Revive a fresh batch of HEK293T cells and use cells at a lower passage number for the experiment.
• Follow all steps from troubleshooting 1 for cell maintenance.
• Cell seeding density is too high or too low. Follow the suggested numbers for seeding.
• HEK293T cells are prone to clumping. Gently resuspend the cells before seeding.

### Problem 3

Excessive cell death after plasmid transfection. Protocol major step 1.

### Potential solution

• Ensure optimum cell health before proceeding with transfection.
• Xfect polymer is cytotoxic. Follow the manufacturer’s protocol to avoid cytotoxicity.
• Plasmid DNA concentration is too high or too low. Balance the ratio of plasmid DNA:Xfect carefully.
• Start monitoring cells 18 hours post transfection to ensure the collection of lysates before excessive cell death occurs.

### Problem 4

In Western blot, protein bands are not detectable.

### Potential solution

• Absence of protein expression or low levels of expression. Revive a fresh batch of HEK293T cells and perform experiment in low passage numbers. Protocol major step 1.
• Transfection is unsuccessful. Check the transfection reagent. Use fresh stock in case of old reagent and follow the manufacturer’s protocol.
• Plasmid DNA is degraded. Prepare fresh plasmid DNA and check its integrity before transfection. Also perform restriction digestion to check the presence of insert in the plasmid.
• Improper protein transfer during Western blotting. Activate PVDF membrane properly and equilibrate the gels, the membranes and blot papers in transfer buffer. Make sure that the transfer apparatus is set up properly. Use a pre-stained molecular weight marker to check the transfer efficiency. Protocol major step 4.
• Antibody dilutions are not effective. Optimize the antibody dilutions for both the primary and secondary antibodies.
• Detection reagent may be contaminated. Use fresh reagent.

### Problem 5

In Western blot, higher-order oligomers are not detected.

### Potential solution

• Prepare a fresh stock of DSP and add it to the lysis buffer just before harvesting the cells. Protocol major step 2.
• Prepare DSP stock solution in tissue culture-grade, high quality DMSO.
• Lower DSP concentration may lead to ineffective crosslinking of proteins. Make sure that the right concentration of DSP is added.
• Shorter incubation time can lead to incomplete crosslinking. Follow the suggested time and procedure for incubation.
• Perform cell lysis and sample preparation properly.
• Avoid confusion while adding the sample buffer with or without BME. Protocol major step 3.
• Resolve the gels properly such that the oligomeric complexes can enter the resolving gel for successful detection. Protocol major step 4.

### Problem 6

In Western blot, there are non-specific bands and a lot of patchy background signal. Protocol major step 4.

### Potential solution

• Increase the duration and number of washes to minimize background signal. Increase the volume of wash buffer.
• Use freshly prepared buffers like transfer buffer, TBST, running buffer etc.
• Optimize the concentration of both the primary and secondary antibodies.
• Blots might be getting overexposed. Reduce the exposure time of the blots.
• Optimize the blocking time and perform blocking step properly.
• Ensure that you are adding the right concentration of DSP. High concentration of DSP can cause non-specific crosslinking and smeared bands or high molecular weight aggregates might appear in the blots.
• Detection reagent may be contaminated. Use fresh reagent.

## Resource availability

### Lead contact

Further information and requests for resources and reagents should be directed to and will be fulfilled by the lead contact, Sannula Kesavardhana (skesav@iisc.ac.in).

### Technical contact

Technical questions on executing this protocol should be directed to and will be answered by the technical contact, Sannula Kesavardhana (skesav@iisc.ac.in).

### Materials availability

This study did not generate any new materials.

### Data and code availability

• Original data for the figure is available upon request.
• This study did not generate/analyze any dataset or code.

## Acknowledgments

We thank Nevan Krogan, Margaret Soucheray, and Ujjwal Rathore from the University of California San Francisco for providing SARS-CoV-2 protein expression clones. This work was supported by funding from the Science and Engineering Research Board (SERB-DST) (EEQ/2021/000274), Department of Biotechnology (DBT) (BT/PR45145/COT/142/24/2022), Indian Council of Medical Research (ICMR) (EMDR/SG/9/2023-2019), (IIRPSG-2024-01-01943), Scheme for Transformational and Advanced Research in Sciences (STARS)-MoE (MoE-STARS/STARS-2/2023-0464), and the Indian Institute of Science (IISc) Start-up grant. S.M. is a recipient of the Prime Minister Research Fellowship (PMRF) scheme in India.

## Author contributions

S.K. developed the protocol; S.M., A.A.D., and S.K. designed experiments, performed cell culture and biochemical experiments, and wrote and edited the manuscript; S.K. provided the guidance and brought the funding.

## Declaration of interests

The authors declare no competing interests.
